# Case Report: Pitfalls in anatomic pathology and clinical oncology: a case of misdiagnosed pulmonary Ewing sarcoma as SCLC

**DOI:** 10.3389/fonc.2025.1635424

**Published:** 2025-11-06

**Authors:** Nagham S. El Waary, Angelo A. Chami, Jawad K. Zrein, Doha A. Houcheimy, Ryan T. Akl, Siham D. Fleifel, Francois G. Kamar

**Affiliations:** 1 Department of Hematology and Oncology, Faculty of Medicine, University of Balamand, Beirut, Lebanon; 2 Division of Hematology and Oncology, Mount Lebanon Hospital University Medical Center, Beirut, Lebanon; 3 Faculty of Medicine and Medical Sciences, University of Balamand, Beirut, Lebanon; 4 Department of Pathology, Lebanese American University Medical Center Rizk Hospital, Beirut, Lebanon

**Keywords:** lung malignancy, pulmonary Ewing sarcoma, immunohistochemistry, molecular testing, multidisciplinary team, misdiagnosis

## Abstract

In oncology, an accurate pathological diagnosis can often mean the difference between cure and failure, potentially determining a patient’s survival. We present the case of a 28-year-old, never-smoking man whose initial diagnosis of small cell lung cancer (SCLC) was confirmed by the anatomic pathology laboratory upon reevaluation, despite initial doubt. This misclassification ultimately led to therapeutic failure following an initial complete remission and likely contributed to the poor outcome after the diagnosis was later corrected to pulmonary Ewing sarcoma. Primary pulmonary Ewing sarcoma is a rare malignancy that is often overlooked in adults. This case underscores not only the striking clinical and histopathological overlap between SCLC and pulmonary Ewing sarcoma but also the potentially fatal consequences of missing key diagnostic red flags, including the patient’s young age, non-smoking status, and atypical clinical course. Through this patient’s journey, we emphasize the importance of multidisciplinary collaboration, the limitations of relying solely on immunohistochemistry, and the critical role of early molecular testing. This case serves as a stark reminder that behind every pathology report is a human life—one that depends on the vigilance, humility, and thoroughness of the medical team entrusted with their care.

## 1 Introduction

In the ever-evolving and dynamic world of oncology and the classification of thoracic malignancies, one must always keep an open and critical mind when diagnosing cases, no matter how straightforward or routine they might appear. An accurate diagnosis can be especially challenging when making a correct classification of thoracic malignancies such as primary mediastinal malignancies or lung cancers, due to the high overlap in histopathological and immunohistochemical features.

Primary mediastinal or pulmonary Ewing sarcoma (PES), an extremely rare malignancy with around 50 cases reported worldwide (1), can be easily misdiagnosed as a case of small cell lung carcinoma (SCLC) because of their similar morphological features—namely, small round blue cell histology (2, 3), a high nuclear-to-cytoplasmic ratio (2), hyperchromatic nuclei (4), abundant mitotic activity (5), or even necrotic and hemorrhagic features (4).

The following article discusses the discrepancies, overlap, and weak points that led to the misdiagnosis of a young gentleman initially thought to have a typical case of SCLC but later found to have an atypical presentation of PES. The delay in accurate diagnosis led to a suboptimal case outcome and the deferment of the correct chemotherapy regimen as well as any radiotherapy or surgical intervention. This case emphasizes the pitfalls in histopathology, the limitations of IHC alone, and the necessity for molecular testing, as well as highlights the importance of a multidisciplinary team approach in oncology discussions.

## 2 Case presentation

Our patient was a 28-year-old, never-smoking man with no significant past medical or surgical history. He initially presented to his local dispensary with gastrointestinal (GI) symptoms, including nausea and vomiting, associated with low anterior chest pain and a “pins and needles” sensation. He was prescribed intramuscular (IM) antibiotics and advised to undergo imaging studies. At that time, his initial computed tomography (CT) scan revealed a 9-cm mass in the left lung.

He presented to our clinic 4 months later with dyspnea and an intractable cough. A chest X-ray showed a widened mediastinum, prompting a repeat CT scan. This time, imaging revealed an 11-cm left hilar mass involving the upper and lower main bronchi. Bronchoscopy with biopsy established a diagnosis of small cell lung carcinoma (SCLC), which was confirmed by immunohistochemistry (IHC). The IHC findings were as follows: + CD56, +cytokeratin, -CD45, -CD20, - TTF1 and p63.

The anatomic pathology department had been consulted several times regarding this diagnosis, since the presentation was not classical given the patient’s young age and never-smoker status.

Staging workup included a normal brain MRI and an FDG PET-CT scan showing an 11x11x10 cm left hilar mass with an SUV of 13 and no evidence of locoregional or distant metastasis. Given the patient’s confined disease, a cisplatin, etoposide, and atezolizumab (Tecentriq) chemotherapy protocol was initiated with concurrent radiotherapy after documenting a major response by FDG PET-CT criteria following the second cycle. This allowed for a significant reduction in the radiation field.

Upon completing the third cycle, another FDG PET scan showed complete remission; therefore, treatment was continued until completion of six cycles. Unfortunately, a post-treatment FDG PET-CT scan showed local recurrence of the disease in the left upper lobe and hilum, with direct invasion into the mediastinum.

**Figure 1:**
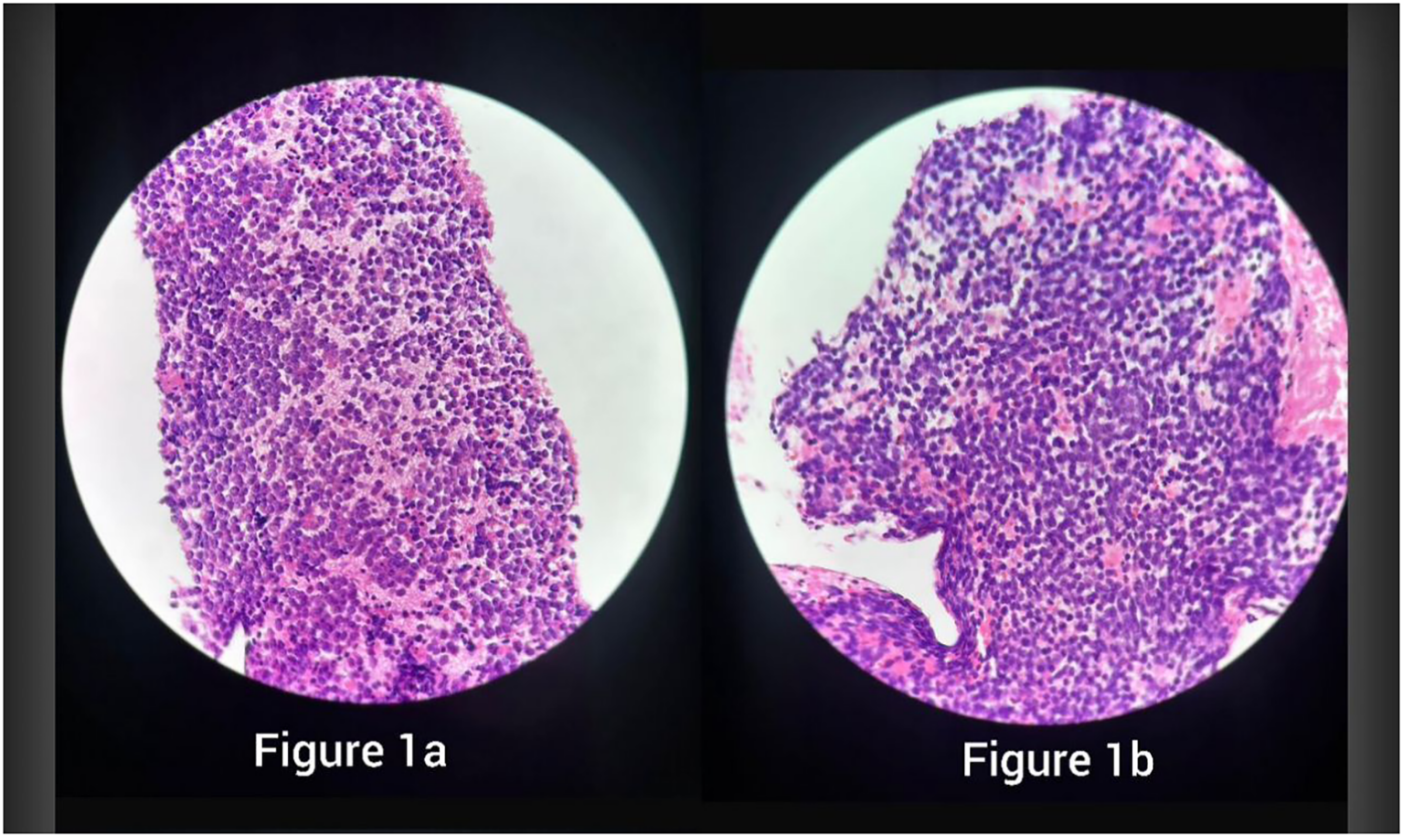
H&E sections showing sheets of small round blue cells with scant cytoplasm and fine chromatin, consistent with Ewing sarcoma, in specimen 1 **(a)** and specimen 2 **(b)**.

**Figure 2:**
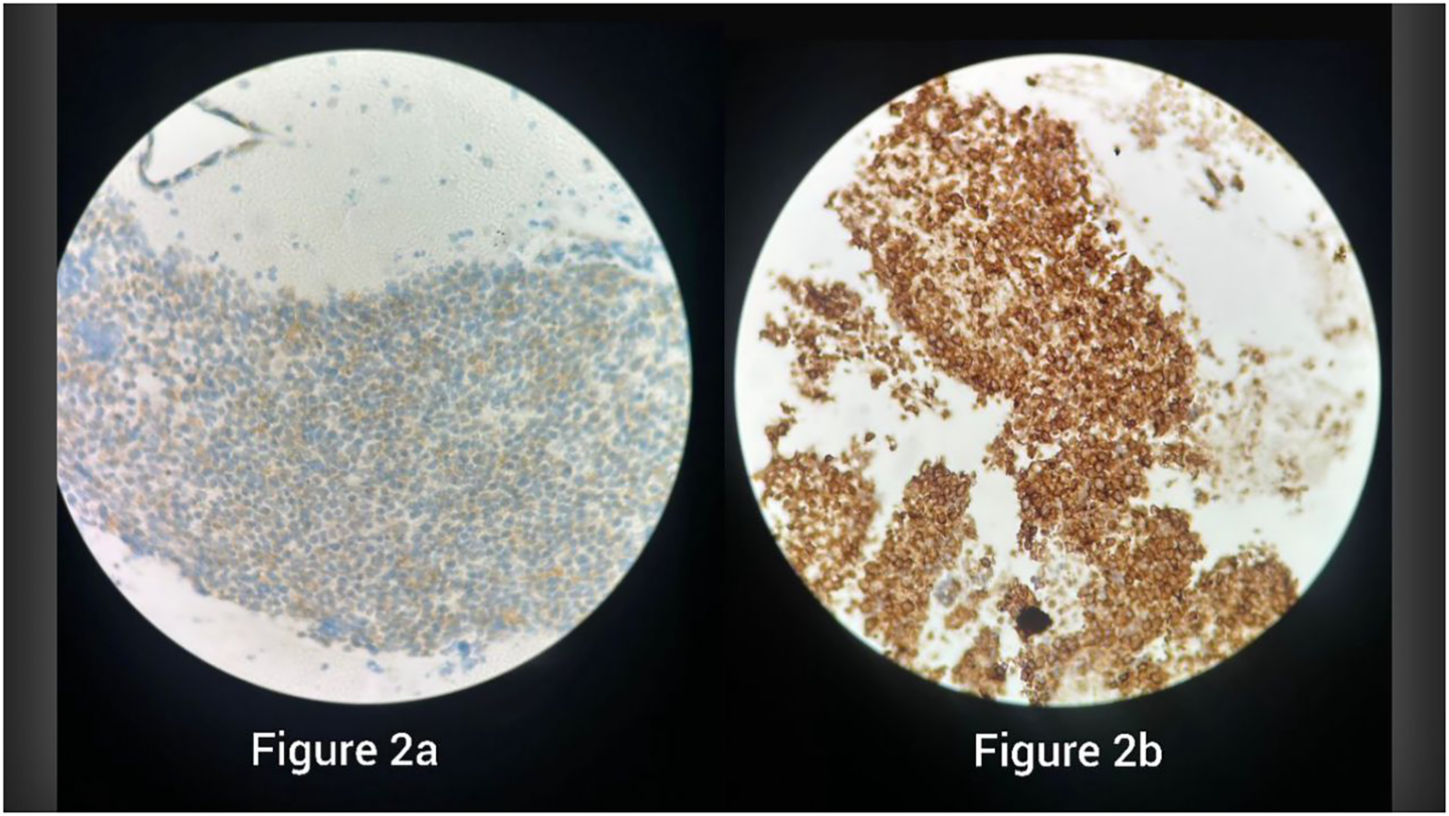
Immunohistochemistry showing strong membranous CD99 positivity in specimen 1 **(a)** and specimen 2 **(b)**, supporting the diagnosis of Ewing sarcoma.

The suspiciously short relapse period prompted further investigation, initially requiring a salvage chemotherapy protocol with irinotecan and carboplatin (Campto-Carbo), as well as scheduling an EBUS biopsy at another site, along with re-examination of the same previously embedded paraffin block. The latter was delayed, but upon completion, immunohistochemical and histopathological analyses, which were sent to a reference pathology department in Lebanon, revealed pulmonary Ewing sarcoma, with CD99 (+), vimentin (+), CD56 (+), CD45 (–), synaptophysin (–), desmin (–), TTF-1 (–), and TLE1 (–). Although NKX2.2 immunohistochemistry and EWSR1 rearrangement studies were not available in our setting to definitively exclude BCOR- or CIC-rearranged sarcomas, the characteristic morphology and diffuse membranous CD99 positivity strongly supported the diagnosis of Ewing sarcoma (**Figures 1**, **2**).

**Figure 3:**
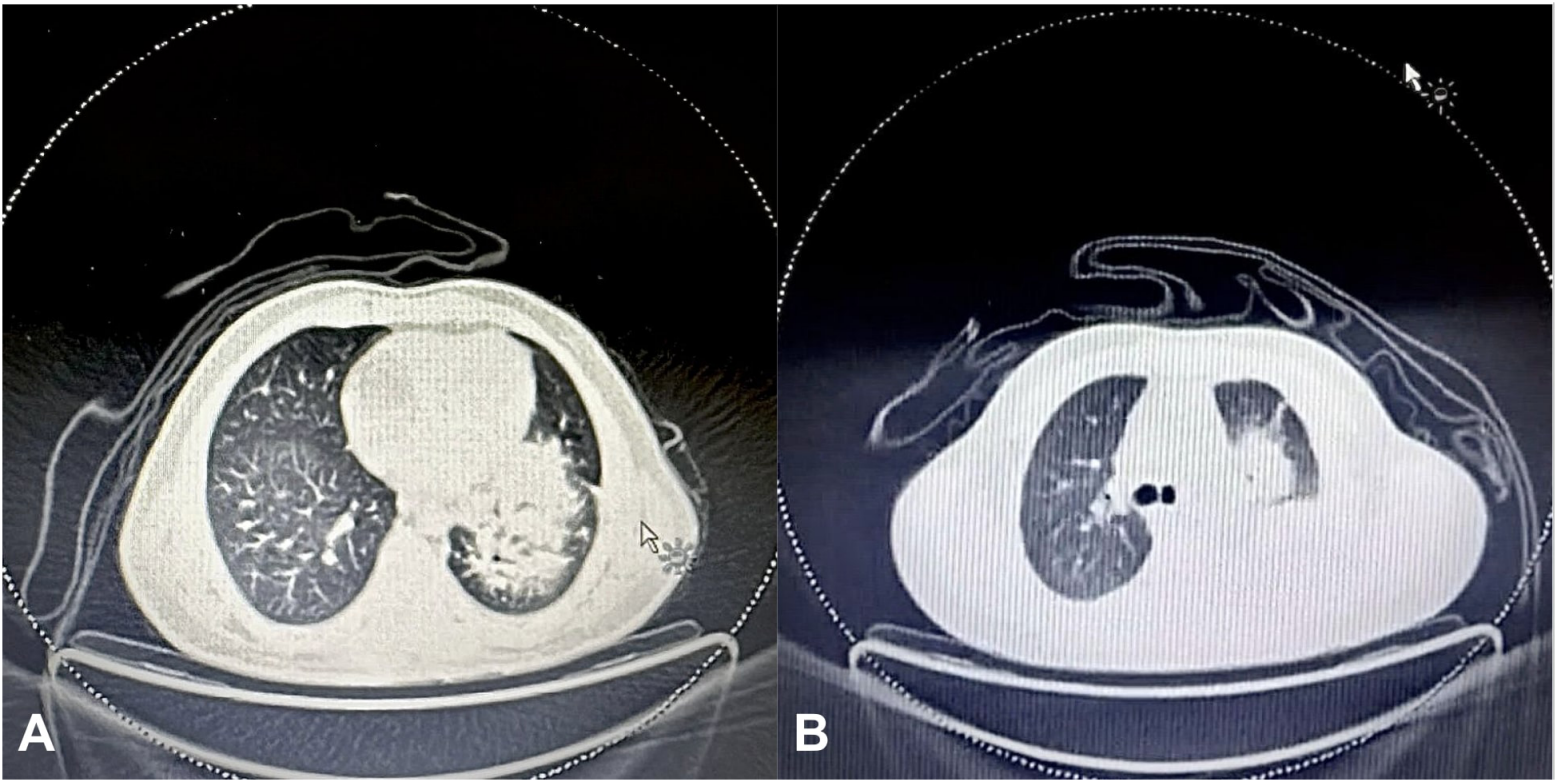
**(A)** Initial FDG PET scan of the chest showing an 11x11x10 cm soft tissue mass in the left lung perihilar region, encasing the left lobar bronchus and extending from the medial pleural surface to the lung periphery. **(B)** Final FDG PET scan showing a marked interval increase in the size of the soft tissue mass in the left lung perihilar region (12x12x8 cm), extending into the upper and lower lobes and reaching the pleural and pericardial surfaces.

**Figure 4:**
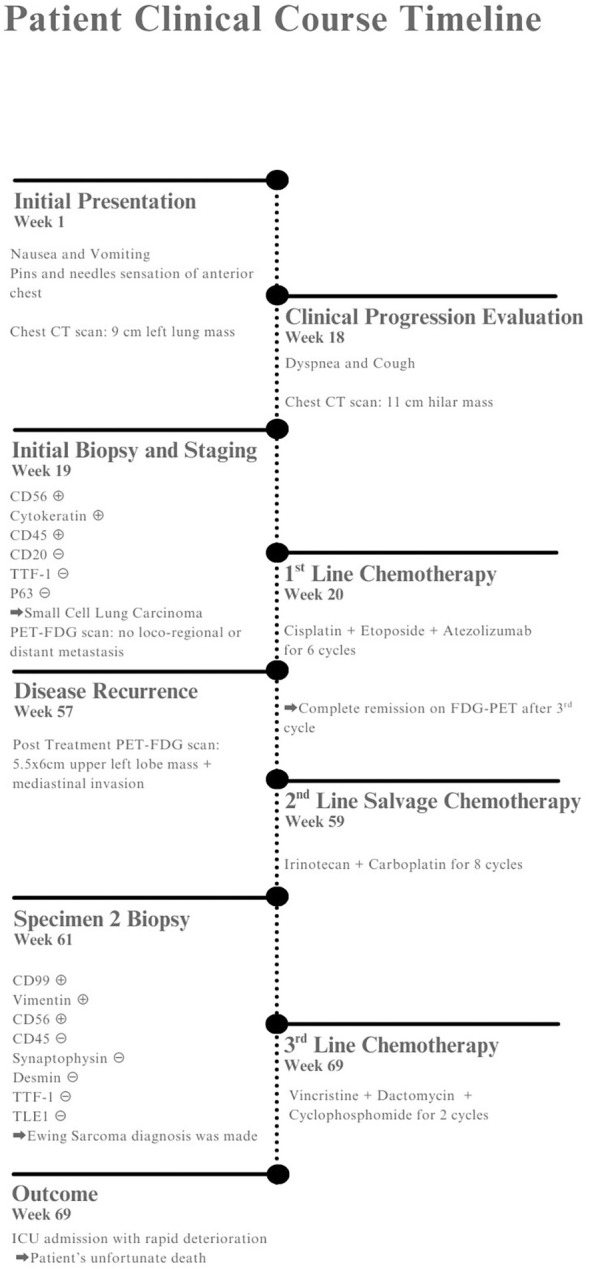
Patient timeline showing clinical presentation, diagnostic workup, treatments, and outcome.

Once these findings were revealed, a repeat PET scan showed significant disease progression **Figure 3**, with a large mass extending from the lower cervical region into the anterior mediastinum and distant metastases to the retroperitoneum and porta hepatis. His condition progressively worsened despite initiation of vincristine, dactinomycin, and cyclophosphamide therapy, ultimately requiring ICU admission. He succumbed to the disease shortly thereafter **Figure 4**.

## 3 Discussion

### 3.1 Why was Ewing sarcoma misdiagnosed as SCLC?

#### 3.1.1 Histopathologic overlap

SCLC and Ewing sarcoma (ES) can both present histologically as small, basophilic cells with granular nuclear chromatin and high mitotic activity on H&E stain. Necrotic and hemorrhagic features are common in both (1, 6). Moreover, although CD56 is the most sensitive marker for SCLC, it is not highly specific, and cases of ES found positive for CD56 have been reported in the literature (7, 8), with the latter being associated with a more aggressive tumor, especially when found in the extraosseous form of ES (8). To add more to the overlap, cytokeratin can be positive in SCLC (6) and, in very rare cases, in ES as well (1).

In the case presented, our misdiagnosis was confirmed at the repeat biopsy by the CD99 and vimentin IHC stains that are sensitive to ES. However, as we will explain further, these stains should not be our sole source of confirmation, as both can show positivity in other types of lung carcinomas, especially in combined forms (9). It is also important to point out that other IHC stains, such as chromogranin A and synaptophysin, are not exclusive to SCLC and that a few cases of extraosseous ES have been reported to display such immunohistological features (1) **Table 1**.

**Table 1:** Immunohistochemical and molecular differences between pulmonary Ewing sarcoma and small cell lung carcinoma.

Marker/feature	Ewing sarcoma	Small cell lung carcinoma
CD99	Diffuse Strong Membranous Positivity	Negative or weak and focal
Vimentin	Positive	Negative
Cytokeratin	Rare (weak)	Diffuse Positivity
NKX2.2	Nuclear Positivity	Negative
FL1-1	Nuclear Positivity	Negative
TTF-1	Negative	Positive in majority of cases
Neuroendocrine Markers (Synatophysin, Chromogranin, CD56)	Negative or rare weak focal positivity	Diffuse positivity for at least one marker
Ki-67 Proliferation Index	High, but variable	Very High (usually ~ 70-90%)
Molecular Confirmation	Most commonly EWSR1-FL1 fusion	Frequent TP53 or RB1 inactivation

#### 3.1.2 Role of molecular testing

Since 1994, Delattre et al. have established that, at the molecular level, fusion of the EWSR1 gene on chromosome 22 with a member of the erythroblast transformation-specific (ETS) family of transcription factors—most commonly *FLI1* on chromosome 11(q24) or ERG on chromosome 21(q22), results in the development of an oncogenic transcription factor that gives rise to Ewing sarcoma in its various forms (10, 11). This translocation remains the most accurate diagnostic standard for Ewing sarcoma and should be considered the gold standard when evaluating these tumors.

More recently, other biomarkers have been identified that can assist in diagnosing ES, although they yet on their own don’t hold the same sensitivity and specificity as the latter.

However, the combination of these biomarkers holds promise in achieving the desired sensitivity and specificity. For example, the NKX2.2 and ZBTB16 genes have been found to be a more sensitive combination when compared to CD99 alone or NKX2.2 and CD99 together. This is because ZBTB16 is upregulated by EWS-FLI1 (12). Moreover, cell-free tumor DNA (ctDNA) containing EWS-FLI1 or EWS-ERG fusions shows not only qualitative value but also quantitative importance in assessing and monitoring tumor burden upon diagnosis and throughout the course of therapy (13).

The lack of molecular testing, either by FISH or NGS, at the initial diagnosis may have led to the unfortunate misdiagnosis of the patient reported in this case. This occurrence has also been reported by Abdelghany et al., who described a case initially misdiagnosed with SCLC and later underwent molecular testing by next-generation sequencing after disease metastasis, only to be found to have primary ES of the lung. Thus, missing out on early molecular confirmation can lead to inappropriate chemotherapy choices, as SCLC and primary ES do not share similar management protocols.

#### 3.1.3 Biopsy site and imaging bias

It is without doubt that the site from which the biopsy is extracted plays a role in the diagnostic approach to the type of tumor, especially given that primary ES of the lung has been reported in the literature. Fedeli et al., in a systematic review of primary ES of the lung, reported 50 cases as of 2023 found in the literature. Moreover, SCLC most frequently presents as a hilar mass with ipsilateral mediastinal lymphadenopathy or direct mediastinal extension, with involvement of the upper and lower lobes being a common presentation and infrequent ipsilateral pleural effusions (14). This added to the bias toward the incorrect diagnosis.

### 3.2 Clinical consequence of misdiagnosis

#### 3.2.1 Inappropriate treatment

The patient’s disease stage prompted initiation of etoposide and a platinum base, a standard of care that has been in use for decades. Our patient was started on cisplatin due to its decreased side effects of myelosuppression and better overall survival in younger patients (6). The patient was also placed on atezolizumab, which has shown modest improvement in overall and progression-free survival (6). The patient additionally received radiation therapy in a VMAT and IGRT fashion between his third and fourth chemotherapy sessions. The aim of management was hopeful, as the patient was young, and complete remission was the goal.

However, SCLC is known to be aggressive, especially in advanced stages, with a 5-year overall survival rate of 40% in early stage, 29% in extensive stage, and 18% in broadly metastatic extensive stage (15). Ewing sarcoma management is considered more intensive and aims for complete remission, as survival rates are high if initiated early. The US-based standard of care, later validated by the Euro Ewing 2012 Phase 3 trial, is the most effective and least toxic, with a shortened duration. The regimen is composed of vincristine, cyclophosphamide, and doxorubicin alternated with ifosfamide and etoposide (16, 17). The chemotherapy regimen can then be followed by radiation therapy or surgical resection, either of which is case dependent.

.

#### 3.2.2 Delayed correct treatment and prognostic implications

Both treatments differ significantly, sharing only etoposide as a common agent, which could explain the false hope created by the partial response to treatment. Thus, the delay in uncovering the true diagnosis led to critical time being lost and disease progression beyond the reach of the standard Ewing regimen, eventually resulting in metastasis and the unfortunate demise of our patient.

Primary pulmonary Ewing sarcoma is an aggressive disease, particularly in the absence of surgical intervention, and carries a very poor prognosis. In the review by Fedeli et al., among 36 reported cases, 14 patients had died by the time of publication, with a median survival of 11.5 months (95% CI, 1.8–25.2). Thirteen patients were alive at a median follow-up of 18 months (95% CI, 14.1–41.1 months), six were alive at 36 months from diagnosis, and three remained disease-free for 48 months (1).

Stork et al., in a retrospective study analyzing nine patients with primary Ewing sarcoma of the mediastinum, reported an overall 5-year survival rate of 64%. Interestingly, patients who underwent local R0 resection for primary, non-metastatic disease achieved a 100% survival rate (18). These findings suggest that high-dose chemotherapy, followed by surgical resection when feasible, could have provided a better prognosis for our patient.

### 3.3 Lessons learned

#### 3.3.1 When to suspect primary Ewing sarcoma instead of SCLC

Recognizing the demographic discrepancy of having a never-smoker young patient with SCLC should have been a warning sign to look deeper into the true etiology of the malignancy. In addition, the poor response and early relapse of the disease were other red flags that should have prompted us to question the primary diagnosis. With Ewing sarcoma being less aggressive and more responsive to dose-intensive regimens of chemoradiotherapy (CRT), precious time was lost in attaining the correct diagnosis, which could have given our patient a better chance of survival.

SCLC’s median age of presentation in both genders was around 68–69 years in 2019 (15, 19), which presents a significant gap when compared with the median age of the rare cases of primary Ewing sarcoma of the lung reported, which was around 30.5 years in both sexes (1). It is also important to examine the risk factors for each malignancy. SCLC occurs in approximately 95% of cases in smokers, with an increased risk in groups that have smoked at low intensity over a long period compared with those who have smoked at higher intensity over a shorter period, even when overall pack-years are the same. The overall risk ranges between 17.1 and 38.6 for 30 years of smoking (20). Only 2%–3% of cases are reported in non-smokers, with the remainder attributed to environmental exposure to carcinogenic materials, mainly radon (20).

As for Ewing sarcoma, no external risk factors are known, and molecular susceptibility to mutations remains the primary cause. Imaging plays a major role in establishing the differential diagnoses of lung tumors, especially given the overlapping features between the two types. Some key radiologic signs of Ewing sarcoma to look out for include a well-circumscribed mass with a heterogeneous appearance (21, 22). Invasion of adjacent structures is rare (21), while ipsilateral pleural effusions and calcifications have been reported (21, 22). On FDG-PET, the malignancy demonstrates increased uptake, which aids in border and invasion detection as well as in identifying bone marrow metastases.

#### 3.3.2 Role of a multidisciplinary team

Accurate diagnosis of complex or unclear pathologies relies significantly on multidisciplinary teams (MDTs), including oncologists, pathologists, and radiologists, particularly in lesions of the lung, where clinical, radiologic, and histopathologic features often overlap in pulmonary masses. In our case, the importance of the MDT is highlighted by the initial pathology report misdiagnosing our patient with SCLC mainly because key IHC markers were not included. When diagnosing Ewing sarcoma of the lung, the central role lies in distinguishing the histological findings of ES from its mimics, as well as identifying essential IHC markers such as CD99, FLI1, and NKX2.2. When morphological features alone are inconclusive or overlapping, these markers are of great importance.

As in our case, the omission of these stains in the initial pathology report delayed the correct diagnosis and, consequently, the correct treatment. This underscores the need for standardized diagnostic protocols to ensure timely identification of the disease. Similarly, the radiologist’s role lies in recognizing the characteristic radiographic features of ES. When imaging modalities reveal aggressive features of malignancy—such as a well-circumscribed mass invading adjacent structures, with signs of pleural effusion and calcifications (21, 22)—especially in young patients, these findings should be emphasized when setting the differential.

In a study conducted by Pang et al. concerning the role of multidisciplinary teams in guiding the diagnosis and treatment of bone and soft tissue tumors, the teams were able to correctly diagnose the type of tumor in 95.42% of cases, compared with a rate of 90.84% when multidisciplinary discussions were not held. Additionally, the team achieved a 100% success rate in identifying relapses (23). Our case aims to highlight the critical role of communication between specialties in achieving an accurate and timely diagnosis.

If an MDT discussion had taken place during our patient’s workup, the pathologist might have considered ES in the differential diagnosis, taking into account the patient’s demographic and clinical presentation, and thus included the necessary IHC markers in the report—leading to an accurate diagnosis from the outset. Consequently, standardizing a diagnostic workflow that mandates a full panel of IHC stains in relevant, highly suspicious cases will improve diagnostic accuracy and prevent delays in initiating the correct treatment.

We also emphasize the inclusion of molecular testing, especially in patient demographics that may present with signs and symptoms of SCLC but do not fit the typical profile, particularly with respect to age and risk factor exposure. Although molecular testing is a more expensive and less accessible option—especially in less developed institutions with limited funding—we cannot rely solely on IHC, as the discrepancies described could lead to similar events in the future. Thus, molecular testing remains a safeguard for accurate diagnosis and should be incorporated early in the diagnostic workflow.

## 4 Conclusion

This case highlights the key points that led to the initial misdiagnosis of Ewing sarcoma (ES) as a typical case of small cell lung cancer (SCLC). Moreover, it reveals areas in the standard diagnosis of thoracic neoplasms that could divert diagnosticians from accurately identifying the pathology at hand, especially when it mimics a more prevalent malignancy both in histopathology and immunohistochemical profile. This prompts a deeper look into molecular diagnostics as a crucial pillar in future pathology confirmations across all oncology scopes and not only in thoracic neoplasms. The early reliance on FISH or next-generation sequencing (NGS) to identify the EWSR1 translocation would have greatly altered the treatment and would optimally have led to a better prognosis.

An essential role must be recognized for multidisciplinary approaches as well as early and accurate molecular studies to improve the outcomes of future cases, establishing the above-mentioned techniques as essentials rather than luxuries.

## Data Availability

The original contributions presented in the study are included in the article/supplementary material. Further inquiries can be directed to the corresponding authors.
